# 2,4-Bis(dimethyl­amino)-1,3,5-trimethyl-6-(nitro­oxy)borazine

**DOI:** 10.1107/S1600536813007484

**Published:** 2013-04-05

**Authors:** Mark A. Rodriguez, Theodore T. Borek

**Affiliations:** a PO Box 5800, MS 1411, Sandia National Laboratories, Albuquerque, NM 87185-1411, USA; b PO Box 5800, MS 0892, Sandia National Laboratories, Albuquerque, NM 87185-0892, USA

## Abstract

In the title compound, C_7_H_21_B_3_N_6_O_3_, the r.m.s. deviation of the borazine ring atoms is 0.019 Å. The dimethyl­amino groups are orientated at 41.80 (7) and 36.43 (7)° with respect to the borazine ring. The nitro­oxy group is almost normal to the borazine ring [dihedral angle = 85.33 (14)°]. The methyl C atom *trans* to the NO_3_ group is displaced by −0.512 (3) Å from the ring plane, whereas the two *ortho*-methyl C atoms are displaced by 0.239 (3) and 0.178 (3) Å.

## Related literature

2,4-Bis(dimethyl­amino)-6-chloro-1,3,5-trimethyl­borazine (II) (Rodriguez & Borek, 2013 ▶) displays a similar structure to the title compound. However, the title compound displays a near planar borazine ring, whereas (II) shows a boat conformation. For further synthetic details, see: Brennan *et al.* (1960 ▶).
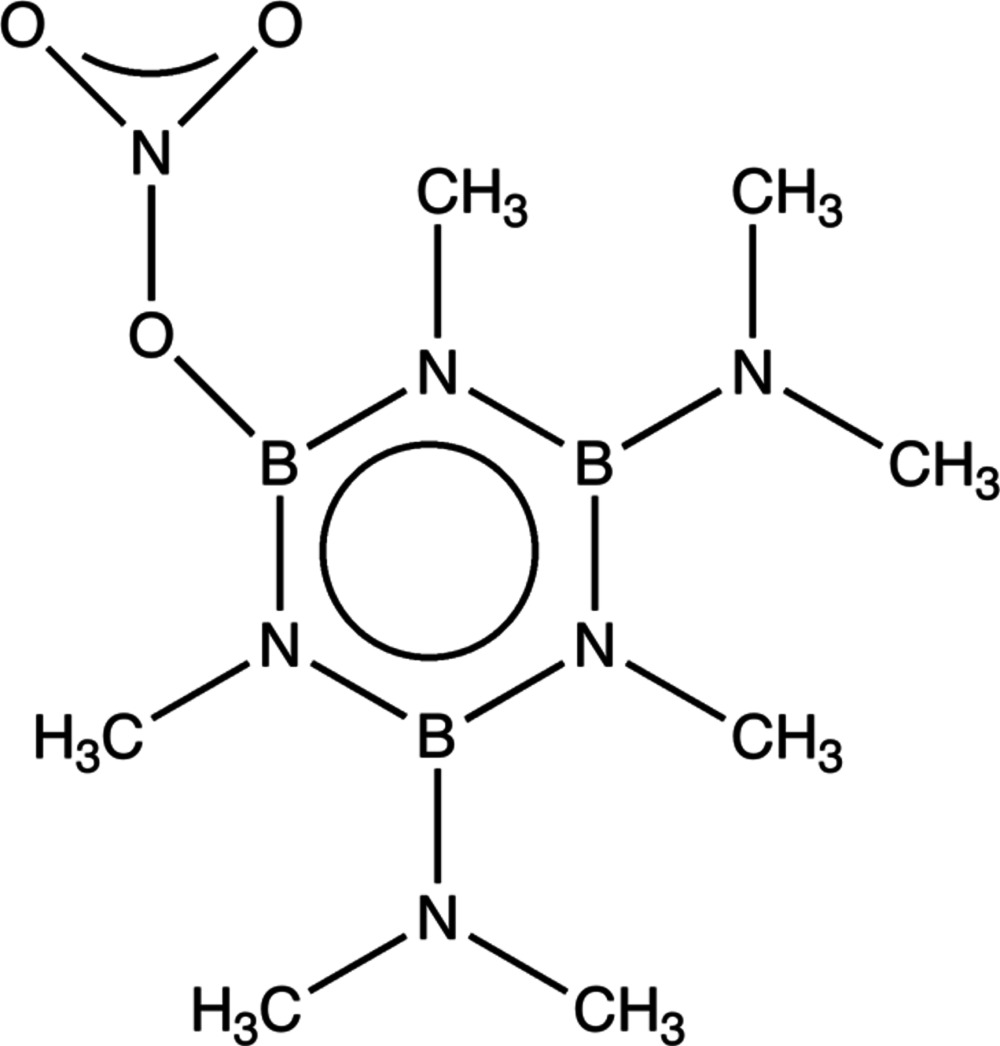


## Experimental

### 

#### Crystal data

C_7_H_21_B_3_N_6_O_3_
*M* *_r_* = 269.73
Triclinic, 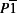

*a* = 8.7017 (15) Å
*b* = 10.2205 (16) Å
*c* = 10.3082 (15) Å
α = 117.624 (2)°
β = 92.371 (2)°
γ = 113.744 (2)°
*V* = 713.5 (2) Å^3^
*Z* = 2
Mo *K*α radiation
μ = 0.09 mm^−1^
*T* = 193 K
0.21 × 0.14 × 0.12 mm

#### Data collection

Bruker APEX CCD diffractometer
Absorption correction: multi-scan (*SADABS*; Bruker, 2005 ▶) *T*_min_ = 0.981, *T*_max_ = 0.990
5210 measured reflections
2515 independent reflections
1754 reflections with *I* > 2σ(*I*)
*R*_int_ = 0.024

#### Refinement

*R*[*F* ^2^ > 2σ(*F* ^2^)] = 0.046
*wR*(*F* ^2^) = 0.124
*S* = 1.03
2515 reflections
179 parameters
H-atom parameters constrained
Δρ_max_ = 0.16 e Å^−3^
Δρ_min_ = −0.24 e Å^−3^

### 

Data collection: *APEX2* (Bruker, 2005 ▶); cell refinement: *SAINT* (Bruker, 2005 ▶); data reduction: *SAINT*; program(s) used to solve structure: *SHELXTL* (Sheldrick, 2008 ▶); program(s) used to refine structure: *SHELXTL*; molecular graphics: *XSHELL.* (Bruker, 2000 ▶) and *Mercury* (Macrae *et al.*, 2006 ▶); software used to prepare material for publication: *SHELXTL*.

## Supplementary Material

Crystal structure: contains datablock(s) global, I. DOI: 10.1107/S1600536813007484/hb7055sup1.cif

Structure factors: contains datablock(s) I. DOI: 10.1107/S1600536813007484/hb7055Isup2.hkl

Supplementary material file. DOI: 10.1107/S1600536813007484/hb7055Isup3.cml

Additional supplementary materials:  crystallographic information; 3D view; checkCIF report

## Figures and Tables

**Fig. 1.**
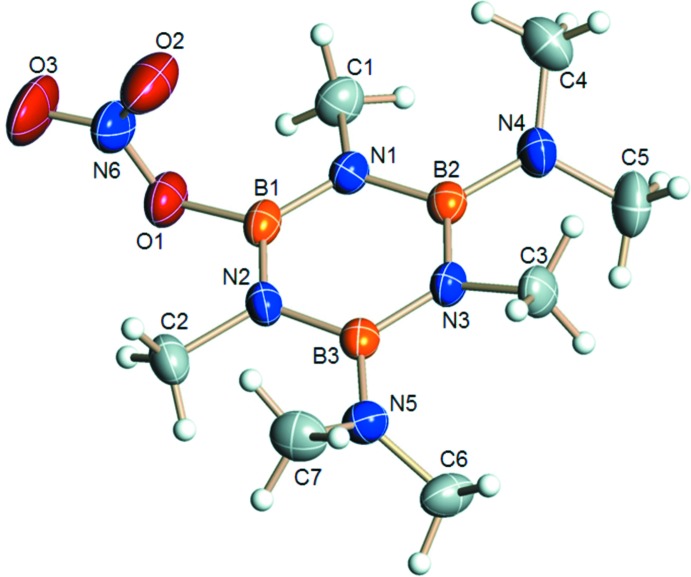
The molecular structure of (I), with atom labels and 50% probability displacement ellipsoids for non-H atoms.

**Fig. 2.**
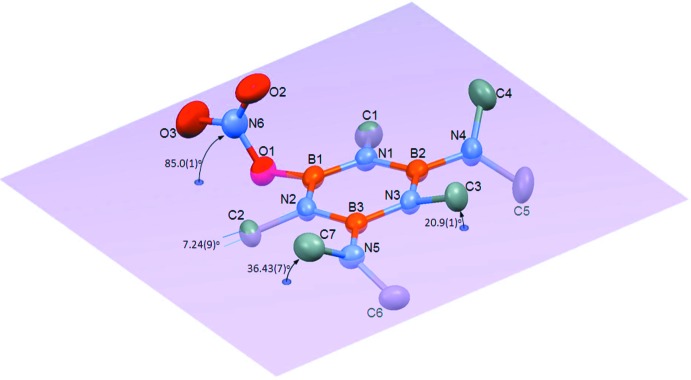
View of molecule (I) with superimposed borazine plane to illustrate devaitions of methyl, dimethylamine, and nitrooxy ligands from the borazine plane. H atoms have been removed for clarity. See text for details.

**Fig. 3.**
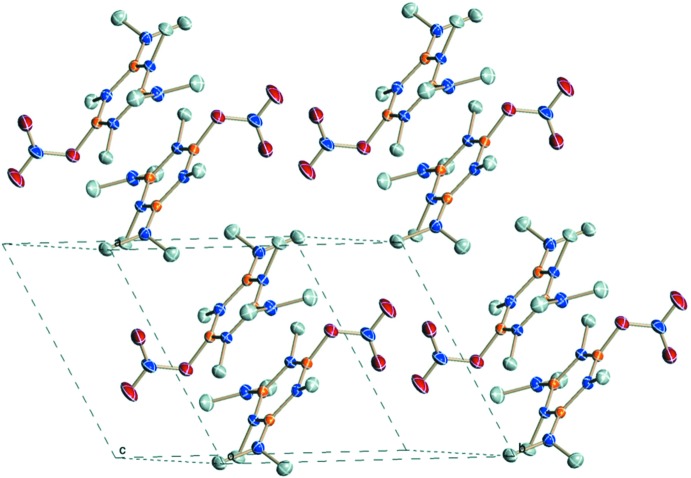
Packing diagram for (I) showing relative orientation of molecules in unit cell. H atoms have been removed for clarity.

## supplementary crystallographic information

### Comment

The 2,4-bis(dimethylamino)-6-nitrooxy-1,3,5-trimethylborazine (I) is a buff-colored solid that has not been previously reported. Figure 1 shows the molecule for this compound as an atomic displacement ellipsoid plot. Bond lengths for the dimethylamine (DMA) ligands, B—N, N—O, and B—O bonds are consistent with expected values. This molecule is very similar to that the previously reported 2,4-Bis(dimethylamino)-6-chloro-1,3,5-trimethylborazine (II); see Rodriguez and Borek, (2013). The difference is merely the exchange of Cl in (II) for a nitrooxy group shown here in (I). The steric nature of the DMA ligands and their proximity to methyl groups bound to the nitrogen atoms of the borazine ring appears to create conditions in the molecule that drive these borazine-bound methyl groups away from the plane created by the borazine ring. Figure 2 shows the molecule of (I) bisected by the plane defined by the borazine ring; the plane is extended through the bound ligands. The view in Figure 2 shows how the C3 methyl, bracketed by the rotated DMA molecules, is displaced upward, out-of-the-plane of the borazine ring (in terms of the molecule orientation in the figure) by an angle of 20.9 (1)°. Likewise, C1 and C2 methyls are displaced downward from the borazine plane by tilt angles of 8.80 (9)° and 7.24 (9)°, respectively. The rotation of the DMA ligands from the borazine plane generates dihedral angles of 41.80 (7)° and 36.43 (7)° for the B2/N4/C4/C5 and B3/N5/C6/C7 DMA groups, respectively. The counter-rotation of the two DMA ligands relative to the C3 methyl is the likely steric mechanism to displace the C3 methyl at a much larger angle compared to the C1 and C2 methyl groups (which are each bracketed by a DMA and the nitrooxy group). The plane defined by the nitrooxy group is nearly perpendicular to the borazine ring, having a dihedral angle of 85.0 (1)° as shown in Figure 2. The O2 and O3 O atoms are terminal and no detection of H atoms was observed in the difference-fourier maps. The molecule is charge balanced as shown.

Figure 3 shows the packing arrangement of the two molecules of (I) within the triclinic unit cell. Additional molecules extending beyond the defined cell are also shown so as to give the viewer a sense of the packing arrangement as it extends in space. Observation of Figure 3 with an eye for symmetry reveals the inversion center present in the unit cell, generating the two formula units per cell (*Z*=2). Based on the absence of any clearly defined donor-acceptor pairs within the structure, there did not appear to be strong hydrogen-bonding interactions within this structure. This was supported by software tests (HTAB) that also indicated the absence of any donor-acceptor pairs. Careful visual inspection of the packing of (I) molecules indicated that the positioning of the terminal O atoms (O2 and O3) were such that they pointed toward H atoms of neighboring methyl groups. Therefore, some weak C—H···O interactions are likely present. However, the distances between the nitrooxy O atoms and neighboring protons exceeded 2.6 Å for C—H···O interactions and the estimated C—H···O bond angles were atypical of hydrogen bonds. Therefore, the packing appears to be dictated by Van der Waals interactions coupled with perhaps weak nitrooxy-methyl interactions.

### Experimental

Compound (I) was obtained using a modification of the published procedure of Brennan, *et al.* (1960). One equivalent of 2,4-Bis(dimethylamino)-6-chloro-1,3,5-trimethylborazine was reacted with one equivalent of silver nitrate in acetonitrile. After stirring the reaction mixture, the solution was filtered to remove precipitated silver chloride, and the solvent was removed using vacuum techniques. This product was then recrystallized from anhydrous hexanes, and then was vacuum distilled (bp 114–116°C at 800 mTorr). The liquid distillate slowly crystallized upon standing at room temperature resulting in a buff-colored solid with a melting point of 68 to 72°C. Crystals formed in this manner were of sufficient quality for single-crystal structure analysis. The product purity was determined by nuclear magnetic resonance (1*H*, 11B, 13C).

### Crystal data

**Table:**

C_7_H_21_B_3_N_6_O_3_	*Z* = 2
*M**_r_* = 269.73	*F*(000) = 288
Triclinic, *P*1	*D*_x_ = 1.255 Mg m^−^^3^
Hall symbol: -P 1	Mo *K*α radiation, λ = 0.71073 Å
*a* = 8.7017 (15) Å	Cell parameters from 200 reflections
*b* = 10.2205 (16) Å	θ = 1.0–25.0°
*c* = 10.3082 (15) Å	µ = 0.09 mm^−^^1^
α = 117.624 (2)°	*T* = 193 K
β = 92.371 (2)°	Irregular, colorless
γ = 113.744 (2)°	0.21 × 0.14 × 0.12 mm
*V* = 713.5 (2) Å^3^	

### Data collection

**Table:**

Bruker APEX CCD diffractometer	2515 independent reflections
Radiation source: fine-focus sealed tube	1754 reflections with *I* > 2σ(*I*)
Graphite monochromator	*R*_int_ = 0.024
ω and φ scans	θ_max_ = 25.0°, θ_min_ = 2.3°
Absorption correction: multi-scan (*SADABS*; Bruker, 2005)	*h* = −10→10
*T*_min_ = 0.981, *T*_max_ = 0.990	*k* = −12→12
5210 measured reflections	*l* = −12→12

### Refinement

**Table:**

Refinement on *F*^2^	Primary atom site location: structure-invariant direct methods
Least-squares matrix: full	Secondary atom site location: difference Fourier map
*R*[*F*^2^ > 2σ(*F*^2^)] = 0.046	Hydrogen site location: inferred from neighbouring sites
*wR*(*F*^2^) = 0.124	H-atom parameters constrained
*S* = 1.03	*w* = 1/[σ^2^(*F*_o_^2^) + (0.0544*P*)^2^ + 0.1672*P*] where *P* = (*F*_o_^2^ + 2*F*_c_^2^)/3
2515 reflections	(Δ/σ)_max_ < 0.001
179 parameters	Δρ_max_ = 0.16 e Å^−^^3^
0 restraints	Δρ_min_ = −0.24 e Å^−^^3^

### Special details

**Table:**

Geometry. All e.s.d.'s (except the e.s.d. in the dihedral angle between two l.s. planes) are estimated using the full covariance matrix. The cell e.s.d.'s are taken into account individually in the estimation of e.s.d.'s in distances, angles and torsion angles; correlations between e.s.d.'s in cell parameters are only used when they are defined by crystal symmetry. An approximate (isotropic) treatment of cell e.s.d.'s is used for estimating e.s.d.'s involving l.s. planes.
Refinement. Refinement of *F*^2^ against ALL reflections. The weighted *R*-factor (*wR*) and goodness of fit (*S*) are based on *F*^2^, conventional *R*-factors (*R*) are based on *F*, with *F* set to zero for negative *F*^2^. The threshold expression of *F*^2^ > σ(*F*^2^) is used only for calculating *R*-factors(gt) *etc*. and is not relevant to the choice of reflections for refinement. *R*-factors based on *F*^2^ are statistically about twice as large as those based on *F*, and *R*- factors based on ALL data will be even larger.

### Fractional atomic coordinates and isotropic or equivalent isotropic displacement parameters (Å^2^)

**Table:**

	*x*	*y*	*z*	*U*_iso_*/*U*_eq_	
B1	0.4283 (3)	0.6785 (3)	0.6075 (3)	0.0344 (5)	
B2	0.2976 (3)	0.5242 (3)	0.7345 (3)	0.0326 (5)	
B3	0.1777 (3)	0.3931 (3)	0.4492 (3)	0.0312 (5)	
N1	0.42950 (19)	0.67279 (19)	0.74152 (18)	0.0326 (4)	
N2	0.3103 (2)	0.5465 (2)	0.46384 (18)	0.0327 (4)	
N3	0.1733 (2)	0.39008 (19)	0.58808 (18)	0.0323 (4)	
N4	0.2920 (2)	0.5148 (2)	0.8690 (2)	0.0424 (5)	
N5	0.0574 (2)	0.2537 (2)	0.30467 (19)	0.0380 (4)	
N6	0.5525 (2)	0.9520 (2)	0.6490 (2)	0.0494 (5)	
O1	0.57519 (18)	0.82087 (18)	0.61309 (18)	0.0487 (4)	
O2	0.4182 (2)	0.9481 (2)	0.6800 (2)	0.0697 (6)	
O3	0.6707 (3)	1.0656 (2)	0.6479 (3)	0.0882 (7)	
C1	0.5786 (3)	0.8066 (3)	0.8791 (2)	0.0450 (6)	
H1A	0.6855	0.8443	0.8486	0.068*	
H1B	0.5931	0.7628	0.9428	0.068*	
H1C	0.5561	0.9014	0.9370	0.068*	
C2	0.3408 (3)	0.5590 (3)	0.3290 (2)	0.0436 (5)	
H2A	0.2795	0.6144	0.3123	0.065*	
H2B	0.2967	0.4462	0.2389	0.065*	
H2C	0.4665	0.6249	0.3464	0.065*	
C3	0.0057 (3)	0.2695 (3)	0.5884 (3)	0.0437 (5)	
H3A	0.0109	0.1674	0.5660	0.066*	
H3B	−0.0904	0.2409	0.5105	0.066*	
H3C	−0.0139	0.3207	0.6889	0.066*	
C4	0.3033 (3)	0.6486 (3)	1.0146 (3)	0.0545 (6)	
H4A	0.3224	0.7458	1.0070	0.082*	
H4B	0.4011	0.6811	1.0936	0.082*	
H4C	0.1941	0.6093	1.0416	0.082*	
C5	0.2485 (3)	0.3612 (3)	0.8680 (3)	0.0579 (7)	
H5A	0.1314	0.3175	0.8816	0.087*	
H5B	0.3342	0.3851	0.9513	0.087*	
H5C	0.2505	0.2775	0.7703	0.087*	
C6	−0.0062 (3)	0.0790 (3)	0.2594 (3)	0.0477 (6)	
H6A	0.0633	0.0735	0.3322	0.072*	
H6B	0.0047	0.0162	0.1572	0.072*	
H6C	−0.1292	0.0297	0.2584	0.072*	
C7	−0.0354 (3)	0.2692 (3)	0.1965 (3)	0.0513 (6)	
H7A	−0.0014	0.3878	0.2379	0.077*	
H7B	−0.1617	0.2060	0.1785	0.077*	
H7C	−0.0057	0.2241	0.1000	0.077*	

### Atomic displacement parameters (Å^2^)

**Table:**

	*U*^11^	*U*^22^	*U*^33^	*U*^12^	*U*^13^	*U*^23^
B1	0.0302 (12)	0.0340 (13)	0.0503 (15)	0.0175 (11)	0.0181 (11)	0.0278 (12)
B2	0.0330 (12)	0.0344 (12)	0.0384 (13)	0.0189 (11)	0.0130 (10)	0.0222 (11)
B3	0.0310 (12)	0.0336 (12)	0.0382 (13)	0.0195 (10)	0.0148 (10)	0.0215 (11)
N1	0.0298 (9)	0.0288 (9)	0.0360 (9)	0.0111 (7)	0.0079 (7)	0.0171 (8)
N2	0.0364 (9)	0.0378 (10)	0.0360 (10)	0.0204 (8)	0.0162 (8)	0.0252 (8)
N3	0.0298 (9)	0.0298 (9)	0.0389 (10)	0.0116 (7)	0.0114 (7)	0.0213 (8)
N4	0.0514 (11)	0.0428 (10)	0.0394 (10)	0.0204 (9)	0.0135 (9)	0.0279 (9)
N5	0.0396 (10)	0.0350 (10)	0.0364 (10)	0.0180 (8)	0.0082 (8)	0.0166 (8)
N6	0.0421 (11)	0.0373 (11)	0.0612 (13)	0.0101 (10)	0.0209 (10)	0.0274 (10)
O1	0.0400 (9)	0.0435 (9)	0.0698 (11)	0.0172 (7)	0.0247 (8)	0.0364 (8)
O2	0.0573 (11)	0.0499 (11)	0.1124 (16)	0.0299 (9)	0.0396 (11)	0.0452 (11)
O3	0.0787 (14)	0.0491 (11)	0.140 (2)	0.0195 (10)	0.0572 (13)	0.0593 (13)
C1	0.0400 (12)	0.0383 (12)	0.0465 (13)	0.0133 (10)	0.0063 (10)	0.0199 (11)
C2	0.0509 (13)	0.0536 (14)	0.0459 (13)	0.0294 (12)	0.0248 (11)	0.0356 (12)
C3	0.0372 (12)	0.0401 (12)	0.0496 (13)	0.0097 (10)	0.0158 (10)	0.0278 (11)
C4	0.0605 (16)	0.0692 (17)	0.0397 (14)	0.0317 (14)	0.0174 (11)	0.0318 (13)
C5	0.0638 (16)	0.0626 (16)	0.0682 (17)	0.0261 (13)	0.0186 (13)	0.0525 (15)
C6	0.0429 (13)	0.0338 (12)	0.0505 (14)	0.0151 (10)	0.0131 (11)	0.0134 (11)
C7	0.0514 (14)	0.0568 (15)	0.0414 (13)	0.0279 (12)	0.0064 (11)	0.0215 (12)

### Geometric parameters (Å, º)

**Table:**

B1—N2	1.405 (3)	C1—H1B	0.9800
B1—N1	1.410 (3)	C1—H1C	0.9800
B1—O1	1.474 (2)	C2—H2A	0.9800
B2—N4	1.434 (3)	C2—H2B	0.9800
B2—N3	1.442 (3)	C2—H2C	0.9800
B2—N1	1.455 (3)	C3—H3A	0.9800
B3—N5	1.430 (3)	C3—H3B	0.9800
B3—N3	1.448 (3)	C3—H3C	0.9800
B3—N2	1.456 (3)	C4—H4A	0.9800
N1—C1	1.478 (3)	C4—H4B	0.9800
N2—C2	1.479 (2)	C4—H4C	0.9800
N3—C3	1.484 (2)	C5—H5A	0.9800
N4—C4	1.453 (3)	C5—H5B	0.9800
N4—C5	1.454 (3)	C5—H5C	0.9800
N5—C7	1.454 (3)	C6—H6A	0.9800
N5—C6	1.457 (3)	C6—H6B	0.9800
N6—O3	1.207 (2)	C6—H6C	0.9800
N6—O2	1.214 (2)	C7—H7A	0.9800
N6—O1	1.316 (2)	C7—H7B	0.9800
C1—H1A	0.9800	C7—H7C	0.9800
			
N2—B1—N1	124.20 (18)	N2—C2—H2B	109.5
N2—B1—O1	117.31 (19)	H2A—C2—H2B	109.5
N1—B1—O1	117.86 (18)	N2—C2—H2C	109.5
N4—B2—N3	122.28 (18)	H2A—C2—H2C	109.5
N4—B2—N1	120.72 (19)	H2B—C2—H2C	109.5
N3—B2—N1	117.00 (18)	N3—C3—H3A	109.5
N5—B3—N3	122.07 (18)	N3—C3—H3B	109.5
N5—B3—N2	121.33 (18)	H3A—C3—H3B	109.5
N3—B3—N2	116.59 (18)	N3—C3—H3C	109.5
B1—N1—B2	118.96 (17)	H3A—C3—H3C	109.5
B1—N1—C1	118.86 (17)	H3B—C3—H3C	109.5
B2—N1—C1	121.61 (17)	N4—C4—H4A	109.5
B1—N2—B3	119.30 (17)	N4—C4—H4B	109.5
B1—N2—C2	118.29 (17)	H4A—C4—H4B	109.5
B3—N2—C2	121.85 (17)	N4—C4—H4C	109.5
B2—N3—B3	123.84 (17)	H4A—C4—H4C	109.5
B2—N3—C3	116.89 (17)	H4B—C4—H4C	109.5
B3—N3—C3	116.69 (16)	N4—C5—H5A	109.5
B2—N4—C4	123.85 (18)	N4—C5—H5B	109.5
B2—N4—C5	123.12 (18)	H5A—C5—H5B	109.5
C4—N4—C5	112.45 (18)	N4—C5—H5C	109.5
B3—N5—C7	123.88 (17)	H5A—C5—H5C	109.5
B3—N5—C6	123.55 (18)	H5B—C5—H5C	109.5
C7—N5—C6	111.94 (17)	N5—C6—H6A	109.5
O3—N6—O2	126.7 (2)	N5—C6—H6B	109.5
O3—N6—O1	115.25 (19)	H6A—C6—H6B	109.5
O2—N6—O1	118.09 (17)	N5—C6—H6C	109.5
N6—O1—B1	115.66 (15)	H6A—C6—H6C	109.5
N1—C1—H1A	109.5	H6B—C6—H6C	109.5
N1—C1—H1B	109.5	N5—C7—H7A	109.5
H1A—C1—H1B	109.5	N5—C7—H7B	109.5
N1—C1—H1C	109.5	H7A—C7—H7B	109.5
H1A—C1—H1C	109.5	N5—C7—H7C	109.5
H1B—C1—H1C	109.5	H7A—C7—H7C	109.5
N2—C2—H2A	109.5	H7B—C7—H7C	109.5
